# GRIEF AND BEREAVEMENT SUPPORT FOR STAFF IN LONG-TERM CARE HOMES: IDENTIFYING BARRIERS AND FACILITATORS

**DOI:** 10.1093/geroni/igae098.3166

**Published:** 2024-12-31

**Authors:** Cari Randa-Beaulieu, Habib Chaudhury, Gloria Puurveen, Theresa Pauly

**Affiliations:** Alzheimer Society of British Columbia, Vancouver, British Columbia, Canada; Simon Fraser University, Vancouver, British Columbia, Canada; University of British Columbia Okanagan, Kelowna, British Columbia, Canada; Simon Fraser University, Vancouver, British Columbia, Canada

## Abstract

Admissions of older adults to long-term care are occurring at more advanced ages with more complex care needs, resulting in shorter lengths of stay before death and an increased demand for a palliative approach to care. However, long-term care staff often lack access to sustainable educational, organizational, and interpersonal support in end-of-life care, and must learn on the job while facing death in the workplace. To address this gap, we conducted a scoping review of literature on grief and bereavement support for long-term care staff. Following PRISMA-Scoping Review guidelines and the six-step framework by Levac and colleagues, social sciences databases were searched and data were analyzed through narrative synthesis and the creation of infographics. Fifty-eight studies met inclusion criteria. Strategies, interventions, and rituals were organized into five domains:1) Cultural Attitudes Toward Death and Unmet Need for Support; 2) Organizational Policy and Practice; 3) Formal Peer-support; 4) Informal Peer-support; and 5) Individual Beliefs and Self-Care Practices influenced by temporal aspects. Identifying barriers and facilitators of grief support for long-term care staff, and hospice and palliative care specialists’ practices, can offer more holistic and effective approaches to long-term care staff wellness to combat burnout and staff turnover. Grief rituals present scalable, holistic approaches fostering long-term care staff wellness that could be explored in future research to collaboratively build long-term care staff capacity for person-centered end-of-life care.

